# Current Challenges and Perspectives for the Use of Aqueous Plant Extracts in the Management of Bacterial Infections: The Case-Study of *Salmonella* *enterica* Serovars

**DOI:** 10.3390/ijms20040940

**Published:** 2019-02-21

**Authors:** Sónia A. O. Santos, Cátia Martins, Carla Pereira, Armando J. D. Silvestre, Sílvia M. Rocha

**Affiliations:** 1 CICECO-Aveiro Institute of Materials, Department of Chemistry, University of Aveiro, 3810-193 Aveiro, Portugal; armsil@ua.pt; 2 QOPNA-Química Orgânica, Produtos Naturais e Agroalimentares, Department of Chemistry, University of Aveiro, 3810-193 Aveiro, Portugal; catiamartins@ua.pt (C.M.); csgp@ua.pt (C.P.)

**Keywords:** antimicrobial activity, *Salmonella* serovars, aqueous plant extracts, bioactive components

## Abstract

Worldwide, foodborne diseases are a growing public health problem. Among the infectious bacteria, non-typhoidal *Salmonella enterica* serovars (NTS) are the major cause of hospitalization and death, and the emergence and spread of their antibiotic-resistance is becoming a worldwide health issue. This, coupled with the restrictions of antibiotics use in agriculture and animal production, calls for alternative approaches to solve this problem. Plant-derived aqueous extracts compounds could provide novel straightforward approaches to control pathogenic bacteria. This review discusses the antimicrobial activity of aqueous plant extracts against *Salmonella* serovars, the possible mechanisms of action involved, which components/structures might be responsible for such activity, and the current challenges for the use of these extracts/components in *Salmonella* infection management and their application perspectives.

## 1. Introduction

Globally, foodborne diseases continue to be a serious public health problem [1]. At the same time, consumers are paying increasing attention to minimally processed food products, with less use of synthetic additives without compromising food safety. The extensive use of antibiotics in human and veterinary medicines and as growth promoters in agriculture animal husbandry and fish farming, although they have been banned from feed in some locations, such as in Europe, is considered to be the major reason for the development of bacterial resistance to antibiotics. Bacteria that originate from animal-based food products frequently carry resistance to a range of antimicrobial agents that are commonly used in humans and it is possible that these resistant organisms, or their genes, can be transferred to humans, either directly via the food chain, or indirectly, as a result of spread of animal manure in agricultural fields. Invasive *Salmonella* infections (typhoid and non-typhoidal) is recognized as one of the main causes of food-borne infections worldwide and it is one of the major causes of morbidity and mortality in Africa and Asia [2,3]. Specifically, invasive non-typhoidal *Salmonella enterica* (iNTS) infections have high incidence in sub-Saharan Africa, presenting mortality rates between 10and 30%, in particular among children and HIV infected adults. The occurrence and prevalence of antibiotic resistant *Salmonella* in humans, animals, and food has been documented [3], presenting a major challenge to the clinical management of these infections, particularly in resource-limited countries. In fact, *Salmonella* has been listed by the World Health Organization (WHO) as one of the antibiotic-resistant priority pathogens, which urgently requires new strategies for infection management [4]. In response to this, the scientific community is devoting efforts to find natural alternative sources of antimicrobial agents that ideally do not promote resistance development. In this context, plants are considered to be an almost unlimited source of bioactive components, and their use as antimicrobial agents has been exploited in different ways [5]. In fact, the relatively low frequency of infectious diseases in wild plants suggests that their natural defence mechanisms can be very effective. It is also important to mention that the development of bacterial resistance towards natural plant products was thus far poorly documented in a very limited number of cases (e.g., for reserpine) [5].

Despite the huge amount of studies in the area, a detailed and critical appraisal of the most important plant extracts with antimicrobial properties, the underlying mechanism of action, the components/structures that may be responsible for such activity, and the identification of the major challenges for the use of these extracts/components in *Salmonella*, therapeutic or prevention is essential. Therefore, to obtain a more comprehensive perspective of the potential use of plant extracts in the prevention or control of bacterial infections, the most relevant studies regarding the evaluation of the antimicrobial activity of plant extracts against *Salmonella* serovars that were published in the (2006–2018) period is critically analyzed in the present review. Despite the diversity of extracts that can be obtained from plants (including essential oils) [6], aqueous extracts were considered as inclusion criteria in order to turn the data comparable, as well as to avoid ambiguous results that arise from dilution problems that some extracts obtained using organic solvents may present in *in vitro* and *in vivo* assays.

It is expected that this revision and the main challenges that were identified in this field would be helpful in the use of more efficient, successful, and straightforward methods to more quickly get to the use of therapeutic natural agents, not only against *Salmonella*, but also against bacteria in general.

## 2. Antimicrobial Potential of Aqueous Plant Extracts

**Figure 1:**
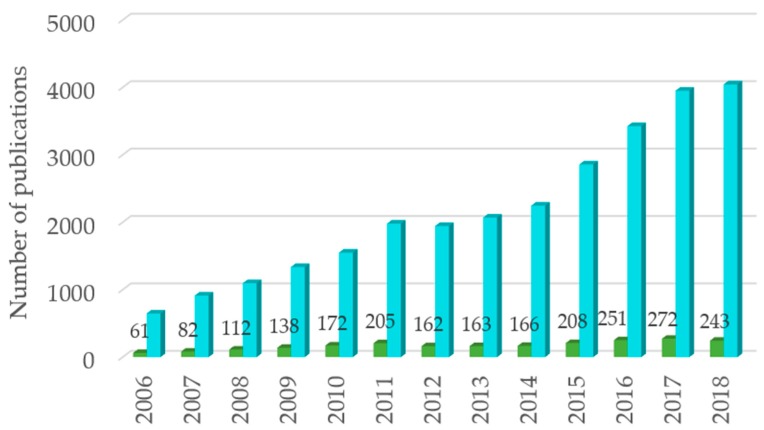
Distribution of studies published in antimicrobial activity of plant extracts, using a search query with keywords 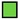
”antimicrobial Salmonella extract” OR “antibacterial Salmonella extract”, and 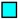
”antimicrobial extract” OR “antibacterial extract” in topic, from 2006 to 2018, via Web of Science^TM^.

**Figure 2:**
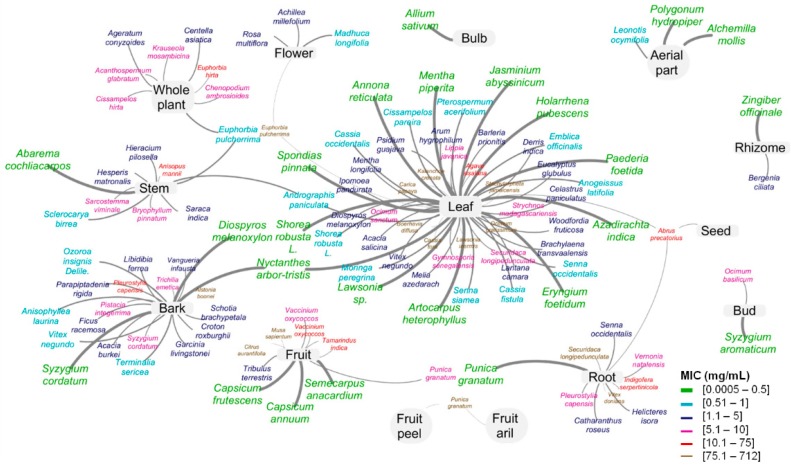
Diagram representing antibacterial activity of aqueous plant extracts on *Salmonella* serovars *in vitro* since 2006, expressed as minimum inhibitory concentration (MIC) (see Table S1 in supplementary material). Grey lines connect the studies between each other through the colored nodes, which represent the plants part used, colored names represent names of plant species, and different colors/line widths represent different MIC ranges (see legend). As MICs increase the size of the letters of the plant, the species name get smaller.

The use of natural products as antimicrobial agents is not new, and a vast range of plants have been used to control infections for centuries. However, in recent years, the increasing demand for natural bioactive components, as a response to a social trend of a healthier diet, as well as to find new components or precursors that are able to decrease the use of antibiotics and to face the resistance development have led researchers to investigate the antimicrobial activity of plants. When considering the last decade (2006–2018), ca. 27,400 studies were published on the topics of antimicrobial or antibacterial extracts, based on the international scientific database ISI Web of Science^TM^ (search query with “antimicrobial extract” or “antibacterial extract”, from 2006 to 2018). This represents a notorious increasing trend, with the number of publications almost doubling in the last five years (Figure 1). It is also worth mentioning, that, during this period, close to 10% of the published studies deal with *Salmonella* serovars. Particularly, ca. 347 studies were published in the same period regarding the antibacterial potential of aqueous plant extracts against *Salmonella* (search query with “*Salmonella* antimicrobial aqueous extract” or “*Salmonella* antibacterial aqueous extract”, from 2006 to 2018). Among these, only 63 studies determined, *in vitro*, the minimum inhibitory concentration (MIC) of the aqueous extracts (Figure 2 and Table S1).

Aqueous extracts have shown antimicrobial effect at concentrations from few µg/mL to mg/mL (0.5 µg/mL to 712 mg/mL), depending on the *Salmonella* serovar tested, the part of the plant used, and plant species. Leaf [7,8,9,10,11,12,13,14,15,16,17,18,19,20,21,22,23,24,25,26,27,28,29,30,31,32,33,34,35,36,37,38,39,40,41,42,43,44,45] has been the most studied part of plants as a source of extracts with activity against *Salmonella*, followed by bark [10,11,14,17,18,19,20,36,37,46,47], stems [11,18,24,25,29,48,49,50,51,52], and roots [7,11,18,19,24,36,37,39,42,53,54,55,56] (Figure 2). The lower minimal inhibitory concentrations (MIC) values are observed for extracts that were obtained from bulbs and leaves; with most of them showing MIC values in the range of 1.1–5 mg/mL. It should be also highlighted that seeds of aqueous extracts often showed no activity [11,17,21,57] or activity only at considerably high concentrations (MIC = 30 mg/mL) [7] against *Salmonella.* A similar behaviour was observed for rhizome extracts [11], with the exception of that from *Zingiber officinale* (MIC = 0.2 mg/mL) [58]. However, it should be noted that these have been among the less exploited plant morphological parts.

Some studies have demonstrated the bactericidal activity of plant extracts against *Salmonella,* rather than only the bacteriostatic action that is provided by MIC [14,35,41,59,60,61,62,63]. Most of these studies have shown minimum bactericidal concentration (MBC) up to four-fold higher than MIC [41,61], with the MBC values ranging from 0.8 mg/mL, for *Shorea robusta* leaf aqueous extract against *S.* Typhi, to 25.0 mg/mL for *Vacicinium oxycoccos* fruit water extracts against *S.* Enteritidis, demonstrating the effectiveness of some plant extracts in inhibiting bacterial growth rather than killing them.

Few studies [64,65,66,67,68,69,70] have proven the antimicrobial capacity of aqueous plant extracts in *Salmonella* serovars *in vivo*, administrating the extracts (doses ranging from 3 to 500 mg of extract/kg of body weight) in animal models before or after *Salmonella* infection. Some of these studies clearly highlight the potential of plant aqueous extracts in the treatment of pathogenic *Salmonella* infections. For example, *Urtica urens* aqueous extract was demonstrated to have a significant effect on mice mortality when administrated in *S.* Typhimurium infected rats at considerably low dosages (3 mg/kg) [70]. Additionally, the administration of *Terminalia belerica* fruits aqueous extract to mice infected with lethal doses of *S.* Typhimurium showed a dose dependent effect, with 83.3 (daily treated with 250 mg extract per kg of body weight) to 100% (daily treated with 500 mg extract per kg of body weight) of the mice surviving after 15 days, while all of the controls have died within seven days [65]. Similarly, Kengni et al. [68] verified that the faeces of infected rats treated with *Harungana madagascariensis* aqueous leaf extract were free of *Salmonella* after 16 days in both male and female animals, even at low extract dose concentrations (25 mg/kg).

Concerning the use of aqueous plant extracts as food preservatives, little is known regarding their action against *Salmonella* serovars in food models. Actually, a single study has been performed incorporating *Puerariae radix* tea aqueous extracts directly in food models [71]. This study demonstrated the growth suppression of *S.* Enteritidis five days after tea aqueous extracts (3–6% (*w*/*w*) or 1–10% (*w*/*v*)) have been incorporated in ground beef and mushroom soup, respectively.

Several studies have also demonstrated the *in vitro* antimicrobial effectiveness of aqueous plant extracts against *Salmonella* serovars after incorporating them in different materials for active packaging applications. As an example, the use of an aqueous extract in the green synthesis of silver nanoparticles showed positive antibacterial activity (MIC = 50 µg/mL) against *Salmonella* Typhimurium [72]. These nanoparticles can be further used, for example, to formulate polymeric materials for food packaging. Similarly, the incorporation of an aqueous cocoa extract (10–20% (*w*/*w*)) in poly(ethylene-vinyl alcohol) copolymer (PEVOH) films resulted in a total growth inhibition against *S.* Enteritidis [73].

The ability of *Salmonella* to form biofilms in abiotic surfaces outside the host, such as in farms, food processing industry, kitchen or toilets, in plant surfaces, or even in animal epithelial cells, contributing to its resistance and persistence, has been documented [74]. However, only a few studies have addressed the ability of aqueous extracts to prevent *Salmonella* biofilm formation [75,76,77]. Vijayan et al. [75] demonstrated, by confocal laser scanning microscopy analysis (CLSM), that silver nanoparticles that were synthesized with an aqueous extract of the macroalga *Turbinaria conoides* were active in controlling the adherence and biofilm formation of *Salmonella* sp., being more active than silver nanoparticles that were synthesized by other methods. The aqueous extracts of two other macroalgae, *Sarcodiotheca gaudichaudii* and *Chondrus crispus* (200 µg/mL), also showed a significantly decrease (3–4-fold) of the biofilm formation of *S.* Enteritidis [76]. In a similar study, a rose aqueous extract that was further submitted to fractionation showed to decrease, up to 7.6-fold (50–300 µg/mL), the *S.* Typhimurium biofilm formation. In addition, during simulated *in vitro* gastrointestinal digestion conditions, it was verified that the gastric digestion did not affect antibiofilm activity, while intestinal digestion significantly reduced the activity [77].

One of the main reasons for the effectiveness of plant extracts to inhibit bacteria growth, and particularly *Salmonella,* is related with the synergistic effects between the extracts active components [78]. Recent studies have reported that the synergism results come from different effects, namely the occurrence of multi-target mechanisms, the existence of components that are able to suppress bacterial resistance mechanisms, the pharmacokinetic or physicochemical effects resulting in enhanced bioavailability, solubility and resorption rate, and the neutralization of adverse effects and the reduction of toxicity [78]. In fact, and despite some controversy, even in antibiotic therapeutics, the combination of two antibiotics or antibiotics with adjuvants has been pointed out as a promising approach, allowing for the reduction of the advance of the resistance of pathogenic bacteria, including *Salmonella* [79,80]. Synergistic interactions between aqueous plant extracts and antibiotics against *Salmonella* have also been observed [81]. *Camellia sinensis* dried leaves (green tea) extract, in combination with nalidixic acid, reflected the inhibition of *S.* Typhi at sub-MIC values. With this combination (C_extract_ = 0.62 mg/mL), nalidixic acid presented a MIC value that was eight-fold lower (32 µg/mL) than when used alone (256 µg/mL), which was observed during all the period of time kill kinetic analysis (8 h) [81]. This strategy could be more deeply exploited, allowing for the expansion of the use of plant extracts in treatment or the prevention of pathogenic *Salmonella* in a near future.

## 3. Mechanism of Action of Plant Extracts. Where Do We Stand?

As complex mixtures of bioactive compounds, aqueous plant extracts (among others) will certainly have several mechanisms of action involved, which could also limit the acquisition of resistance by bacteria, as mentioned before. The precise mechanism or target of most plant bioactive components against bacteria, in general, is not yet elucidated, however there are several mechanisms that have been suggested to be involved, namely the disruption of pathogen membranes, interruption of DNA/RNA synthesis and function, interference with intermediary metabolism, induction of coagulation of cytoplasmic constituents, and the interruption of normal cell communication (quorum sensing, QS) [1,5]. In addition, the antimicrobial activity of natural bioactive components can be also related with their capacity to activate cells of the immune system, as well as to promote the increase of beneficial bacteria in the gut [82]. It has been also proposed that, in the case of phenolic compounds, which are commonly present in aqueous plant extracts, their antimicrobial activity may be also related with their capacity to chelate iron [83], which is required for almost all bacteria survival, including *Salmonella*, for which an increased growth and virulence with iron availability has been described [84]. Notwithstanding, most of the literature regarding the antimicrobial action of bioactive compounds, in general, points out that their primary target site is the cytoplasmic membrane, affecting its structure and integrity, permeability or functionality in different ways [1,85,86,87], including the efflux system. In fact, it has been suggested that plant extracts with activity against Gram-negative bacteria, due to the innate multidrug resistance of these bacteria, may contain inhibitors of efflux pump in their composition [85]. In addition, QS inhibition has been also described as one of the most promising mechanisms of action of natural bioactive compounds against multidrug resistant pathogens, since it was discovered that pathogenic bacteria employ QS to regulate their virulence [85]. It has been pointed that ideal QS inhibitors should be low molecular weight compounds, be able to decrease the expression of QS-controlled genes, and being chemically stable to resist to the metabolic and disposal processes of the host organism, thus making natural compounds very promising [1]. Additionally, most of the caused events leading to antimicrobial action may be inter-related, being affected as a consequence of other targeted mechanisms.

Little is known about the mechanism of action of bioactive compounds that are present in aqueous plant extracts against *Salmonella*. A study with an aqueous yerba mate extract against *S.* Typhimurium demonstrated a major change on central carbon metabolism, a reduction of catalase activity, and no change of membrane integrity [88]. However, the absence of a detailed characterization of the extract used, or the use of a previously characterized extract, hampers the establishment of correlations between the identified mechanisms of action and the extract composition. Notwithstanding, the abundance of saponins and phenolic compounds in yerba mate is well documented [89] and, when considering their high polarity, they should be present in aqueous extracts. In this line, valuable information can be taken from studies involving polar standard components. However, and due to their recognition as one of the most promising (polar) natural bioactive components, phenolic compounds have been the most studied group in this sense.

Scanning electron microscopy (SEM) images of *Salmonella* Choleraesuis that was treated with phenolic compounds indicated the damage of the bacterial cell barrier structure, causing the leakage of cytoplasmic components, such as proteins, nucleic acids, among other compounds [90]. The cells of the bacteria treated with xanthohumol, for example, were shown to be empty. The QS inhibition from natural bioactive components against *Salmonella* has been also reported by several authors [91]; however, it should be highlighted that the assays that were used to evaluate this action have been done with bacteria models, such as *Chromobacterium violaceum* (an opportunistic bacteria), which would compromise the extrapolation of such conclusions to *Salmonella*. A review from Rempe et al. [92] compiled the data regarding the mechanisms of action of several phenolic compounds against different bacteria, including *Salmonella* serovars. Interestingly, the mechanisms of action of the different tested components seem to be grouped by their structure type. Those with a single aromatic ring were shown to act by disrupting cell membranes, with phenolic derivatives showing a reduction of unsaturated fatty acid content, while flavones and flavonols displayed the inactivation of the Type III secretion system of *Salmonella* Typhimurium.

In addition to the lack of studies regarding the evaluation of the specific mechanisms of action of the active components from aqueous plant extracts, most of the studies target specific mechanisms, instead of exploring all of the possibilities, which limit them for specific mechanisms of action. In fact, and besides the synergic effect that could arise from a complex plant extract, the possibility of a single component acting through distinct mechanisms against *Salmonella*, should not be discarded, which is in line with reported results for other bacteria [92].

Finally, it is important to point out that, in general, the antimicrobial potential and mechanism of action of bioactive compounds will not only be modulated by the features of target microorganisms. Actually, they depend on a network of extrinsic and intrinsic factors, namely the environment where the antimicrobial action is exhibited, i.e., redox potential of the environment surrounding, moisture content, hydrophilicity, temperature, pH and acidity, availability of certain basic nutrients for growth, and maintenance of metabolic functions, among others [1]. Therefore, the conditions in which these studies are performed are determinant in the correct interpretation of these mechanisms.

## 4. Looking for Structure-Antimicrobial Activity Relationship

The effect of the different natural compounds structures and action mechanisms that are behind the antimicrobial activity of aqueous extracts against *Salmonella* is not yet fully elucidated. The major concern in this elucidation is related with the complexity of natural extracts, together with the frequent lack of a detailed knowledge about their composition. Some authors have assigned the antibacterial activity of aqueous extracts to different families, such as saponins [62], alkaloids, steroids [59], carbohydrates and reducing sugars [60], and phenolic compounds [93,94,95], only based on colorimetric methods. Notwithstanding, due to the high polarity of aqueous extracts a higher abundance of phenolic compounds is expected, including phenolic and hydroxycinnamic acids, flavonoids, and tannins, which are well known antibacterial agents. Somewhat surprisingly, only two studies [61,96] amongst the high number of works concerning the antibacterial activity against *Salmonella* of aqueous extracts (Table S1 and references therein) have analyzed the phenolic composition of the studied extracts in detail, namely by high-performance liquid chromatography (HPLC) and identifying and quantifying the major components, namely phenolic acids, flavan-3-ols, and flavonoid glycosides. The *Vaccinium oxycoccos* fruit [61] and *Alchemilla mollis* aerial parts [96] aqueous extracts showed considerably different MIC values against *S.* Enteritidis, namely 12.5 and 0.125 mg/mL, respectively. These extracts showed similar phenolic acids composition (among simple, cinnamic acids, and their derivatives) and contents (2.3–2.5 mg/g of extract), although *Alchemilla mollis* extract presented a higher content on simple phenolic acids. However, the main difference was verified in the high abundance of methyl gallate (12.18 mg/g of extract) and luteolin-7-*O*-glucoside (11.63 mg/g of extract), which may be responsible for the considerably higher MIC value that was obtained for *Alchemilla mollis.* In the same study, Stobnicka and Gniewosz [61] compared the aqueous extracts from *Vaccinium oxycoccos* fruit and pomace, which showed the same MIC value against *S.* Enteritidis, while against *S.* Typhimurium, the pomace extracts presented a MIC value that was two-fold higher than fruit extract. Although both of the extracts have shown to be composed by the same constituents, the pomace extract presented phenolic acids and flavonol contents that were approximately, respectively, three-fold and two-fold higher than fruit extract. Therefore, the phenolic profiles that were observed in these studies did not explain the differences or similarities observed in the antibacterial activity of the different extracts.

A third study also reported the HPLC analysis of the aqueous extracts used [46], namely *Libidibia ferrea* and *Parapiptadenia rigida* barks and *Psidium guajava* leaves extracts; however, although complex chromatograms have been obtained, only gallic acid and catechin, even not being the major components, were identified and quantified. Even so, different contents were observed for the three aqueous extracts, although the same MIC value has been obtained (5 mg/mL), which demonstrates that the components quantified were not, at least, uniquely responsible for the antimicrobial activity. In fact, the structural diversity of phenolic compounds structures, together with synergism effects that may occur, hampers the prediction of the structural features of the extract components that are responsible for the activity.

There is also a lack of studies on the comprehension of structure-activity relationship with individual components, even for bacteria in general. One of the first assumptions is that the effectiveness of bioactive compounds generally increases with their increasing lipophilicity, which is related with their ability to interact with the cell membrane [1]. In fact, it has been reported that the activity of phenolic acids against both Gram-positive and Gram-negative bacteria increases with the presence and increasing length of the alkyl chains [97]. In general, it has been reported that the electron distribution, which is affected by position and number of hydroxyl groups and double bonds, is the major factor affecting the antimicrobial activity on phenolic compounds [92]. Notwithstanding, there are other features of natural compounds affecting their activity, such as the hydrogen-bonding or covalent bond formation capacity, which is related with their ability to bind cell walls, disintegrate them, or even to compete with inhibition action mechanisms.

When considering our case study in particular, the antibacterial activity of several phenolic benzaldehydes and benzoic acids against *Salmonella enterica* were shown to be more dependent on the substituents (–OH and –OCH_3_) positions than on their number [98]. The presence of an aldehyde (CHO) instead of carboxylic group (COOH) was also shown to be a structural feature to drastically increase the activity against this strain.

When considering flavonoids, these were shown to be more active against different *Salmonella enterica* serovars when used alone rather than in glycosylated forms [90], which is in line with the tendency described before that relates increasing activity with increasing hydrophobicity. Nevertheless, in a study using the Enteritidis serovar, the combination of flavonoid glycosides with flavonoid aglycones was shown to drastically increase the antibacterial activity [99], once more highlighting the complexity of the synergic effects that might occur in a natural extract.

Other studies have shown the complexity to predict a relation between structure and the activity against *Salmonella* serovars of phenolic compounds rich extracts. An antimicrobial activity study comparing the different hydroxycinnamic acids, flavonoids, and anthocyanidins reported inhibition against *Salmonella enterica* only for hydroxycinnamic acids [100], demonstrating that the antibacterial activity is not only related with the lipophilicity. Costabile et al. [101] investigated the antibacterial activity of different ellagitannins, gallotaninns, condensed tannins, and flavanol gallates fractions that were obtained from different plants against *Salmonella* Typhimurium and no relevant differences were verified between their activities. A study on gallotannins antimicrobial activity showed a positive effect of the degree of galloylation on the activity against *Salmonella* Typhimurium up to seven galloylglucopyranoses units, from which (8 to 10) the activity decreased [102]. This may be explained not only by their lower hydrophobicity, but also by the higher molecular weight that may limit their penetration in the membrane wall.

Thus, the antimicrobial activity of natural components is strongly dependent on the backbone structure, number, position and nature of substituent groups, presence of glycosidic linkages, and alkylation of OH groups. Although, in general, the activity against *Salmonella* tends to increase with the components lipophilicity, the possible synergic or antagonistic effects taking place in complex extracts, together with the innumerous possibilities of mechanisms of action makes it difficult to predict a direct relation between the aqueous extracts composition (and therefore the different components structure present or prevalent), and their activity against *Salmonella*.

## 5. Current Challenges for Development of New Antimicrobials from Plant Extracts

Despite the high number of studies claiming antimicrobial activity for aqueous plant extracts against *Salmonella* (Figure 2 and Table S1), there are several challenges that need to be overcome for the development of new antimicrobials from aqueous plant extracts. One that can be highlighted is to consider only the extracts presenting low or moderate MIC values, not claiming activity for extracts used at high concentrations. This should be the first effort made by the scientific community in an integrated strategy to develop antimicrobial agents from natural sources. Therefore, as already suggested by Ríos and Recio [103], the activity of extracts having MIC values higher than 1 mg/mL or 0.1 mg/mL for isolated compounds should not be considered, while those that inhibit the growth of microorganisms in concentrations below 100 μg/mL and 10 μg/mL, respectively, should deserve the utmost attention and additional research may be done. In this context, the number of aqueous extracts presenting activity against *Salmonella* serovars deeply decrease from 128 to only 45 in the period of 2006–2018 (Figure S1).

The most promising extracts should also be tested regarding their MBC, because it gives information regarding the potential of an extract to kill bacteria rather than just to inhibit their growth. This could be quite important, because, on the one hand, the bactericidal potential hinders the possibility of antimicrobial resistance, and on the other hand, the MIC is not indicative of antimicrobial potential against non-growing bacteria. These bacteria can revert to a growing state besides that they can undergo mutagenic modifications, promoting the resistance against antimicrobial agents [104]. Additionally, it is recommended that the antibiofilm activity may also be addressed in future works, since the biofilms formation has been recognized as the major reason for increasing the survival of bacteria, and particularly of *Salmonella* [74], in adverse environmental conditions, thus contributing to its resistance.

The detailed knowledge of the extract composition is another important task in order to allow the reproducibility of the activities in further works. For those promising extracts, it becomes crucial to understand firstly the plant conditions (geographic origin, season of collection, and plant pre-treatments) that maximize the target compounds, and at the same time allow for obtaining reproducible extract compositions, which are essential for an effective application. Additionally, the knowledge of the active compounds that are present in an extract is also crucial to design and optimize efficient, sustainable, and environmental friendly technologies of fractionation. Even though aqueous extracts are green (but not necessarily sustainable) themselves, the improvement of the effectiveness/activity will certainly require extracts fractionation into enriched fractions or isolated components. Therefore, the detailed analysis of the plant extracts composition, together with the use of alternative and/or more selective extraction or fractionation methods, also when considering the use of alternative solvents (e.g., biocompatible ionic liquids and/or deep eutectic solvents and their aqueous solutions in particular), will certainly contribute to the effective exploitation of bioactive plant extracts in the future.

Another challenge in the development of antibacterial therapeutic agents or food preservatives from plants deals with the wide possibility of synergism or antagonism effects due to the complexity in extracts composition. Therefore, the development and use of methodologies to understand or even to predict which components/mixtures are responsible for a specific activity are of extreme importance. Biossay-guided fractionation or, more recently, synergy-directed fractionation, in which a known active compound from the original extract is added to the collected fractions and the activity tested, have shown to be effective [105]. However, these assays require the isolation of pure compounds, and sometimes the extraction/fractionation process is guided by the components abundance and the ease of separation. The recent development of “biochemometric” assays, which use statistical modelling to predict and correlate the metabolomic profile of extracts and their bioactivity has gained much attention [106]. Although this methodology has not yet been applied to the analysis of plant extracts activity against *Salmonella*, this could be a turning point in the knowledge and comprehension of the relation between the active components structure and its activity.

The development of antibacterial agents for oral therapy, in particular, from aqueous plant extracts, will also require the use of methodologies that consider the effect of digestion on the stability and therefore on the extracts activity. An estimative of bioavailability and bioactivity and a thorough understanding of changes that occur during digestion (such as mechanical action, enzymatic activities, and altered pH) is crucial in evaluating the bioaccessibility, as only bioavailable compounds will exert fully their potential beneficial effects [107]. *In vitro* assays that are able to simulate digestion have been already developed, which dispense the use of lengthy, costly, and ethical controversial animal or human studies. Even though, few studies can be found on the *in vitro* effect of digestion on the activity of aqueous plant extracts against *Salmonella* [77]. Notwithstanding, some limitations can be also addressed to these *in vitro* studies, particularly the impossibility to simulate oral, gastric, and intestinal conditions, besides the interactions between the ingested compounds. Therefore, a final clinical study will always be required to confirm the potential of any extract, enriched fraction, or isolated compound.

There are also several ways to enhance the antimicrobial activity of an agent or even to allow for their controlled delivery in a desired specific site, which will increase their efficacy that must be considered, such as the use of nanotechnology, hydrogel formulation, or bio-adhesive technology.

Interestingly, the most promising extracts against *Salmonella*, i.e., those presenting MIC values between 0.001 and 0.5 mg/mL, such as the case of *Lawsonia* sp. (plant) [15] and *Jasminum abyssinicum* [27] leaves, *Polygonum hydropiper* aerial parts [108], or *Syzygium aromaticum* bud [109] extracts (Figure S1 and Table S1), have not yet been tested *in vivo*, nor incorporated in materials for controlled release, as already performed for other extracts [64,65,66,72,110], which would certainly improve their potential application against these bacteria. On the other hand, the exploitation of the most active extracts will also compromise the future of the discovery of natural antimicrobials. Therefore, an integrated partnership between the scientific community and industries will be quite crucial before any natural component or extract could be used in antimicrobial therapeutic or as food preservative against pathogenic *Salmonella*.

Few studies have studied the toxicity of aqueous plant extract active against *Salmonella*. *In vitro* cellular toxicity studies have been performed using human erythrocytes [69] or human intestinal Caco-2 cells [73]. Results using the haemolysis assay suggested that *Withania somnifera* leaf aqueous extract that was tested in concentrations ranging from 0.5 to 2.0 mg/mL (considerably higher than the estimated MIC = 0.25 mg/mL) causes least haemolysis of the erythrocytes as compared to a standard antibiotic (chloramphenicol) [69]. It was also demonstrated that cocoa aqueous extract, which was shown to promote activity against *S. enterica* when incorporated in ethylene–vinyl alcohol copolymer films, does not produce a synergistic effect with H_2_O_2_ in Caco-2 cell damage in concentration ranges from 100 to 300 µg/mL [73]. Further studies regarding the *in vivo* toxicity of aqueous extracts must be developed, although their low toxicity has been already reported [67,68]. Particularly, in studies that were performed with mice administered with *Vitellaria paradoxa* leaf aqueous extracts at doses ≤4 g/kg, no behavioural changes were observed for gathering, locomotion, reaction to noise, state of the tail, consistency of the excrement, and mortality [67]. However, in a different study, lower doses (>100 mg/kg) of *Harungana madagascariensis* aqueous leaf extract that was administered to infected rats induced hypercholesterolaemia and liver damage, but no effect on kidney functions was observed [68], pointing out the need of carefully addressing extracts toxicity. An aqueous extract of *Euphorbia prostratra* also showed toxicity at doses (≥73.48 mg/kg and ≥122.71 mg/kg for female and male, respectively) significantly higher than those required to treat *S.* Typhimurium-infected rats (26.34 mg/kg) [64]. Although some of these extracts have been considered as practically non-toxic, such as the case of the lately mentioned *Euphorbia prostratra* aqueous extracts, for which the median lethal dose (LD_50_) in mice was verified to be 23.2 g/kg and 26.4 g/kg for female and male, respectively [111], particular attention has to be paid for specific organ/function injuries. Therefore, more studies have to be done, in particular, concerning the chronic toxicity, mutagenicity, and carcinogenicity. Finally, the toxicity of the most promising aqueous extracts in humans has also to be evaluated in order to possibly turn their applicability in *Salmomella* infections management.

## 6. Concluding Remarks and Future Perspectives

Plant bioactive components have historically been a promising source of bioactive compounds, nowadays still being the object of several studies in the search for new pharmacological or bio-preservatives components, including to treat or prevent infections with pathogenic bacteria as *Salmonella*. Leaves have been among the major part of plants exploited for antimicrobials against these bacteria, also showing to be the most promising. However, one major concern in the revisited literature is the lack of strategies to unambiguously identify the active components and the possible synergetic or antagonist effects governing their antibacterial activity. Additionally, little is known about the relationship between the components structure and their activity against *Salmonella*, although the lipophilicity has shown to be a positive effect. Recent developments in metabolomics may play a key role in the identification and effective application of new occurring natural antimicrobials. The knowledge of the mechanisms of action of natural antimicrobial agents is even more elusive, as host organisms typically employ several defensive strategies that may complement, enhance, and enable the activities of other contributors. Further, for the development of therapeutic natural agents, in particular, the use of digestion models has been developed and should be applied, which provides valuable scientific insights into the assessment of components bioaccessibility, the development and testing of drug formulations, and the understanding of microorganism fate under digestive conditions. However, even using the sophisticated *in vitro* models that were developed for this purpose, it is still impossible to fully mimic the overall digestive parameters *in vivo*.

The use of emerging technologies, such as nanotechnology and bio-adhesive technology and materials, namely hydrogel formulations and active (and sometimes also edible) packaging materials in combination with plant bioactive components, should be considered in order to enhance the effectiveness of plant antimicrobial components.

The use of aqueous plant extracts or isolated components in *Salmonella* therapeutic in combination with antibiotics should also be considered (as for other bacteria) as a more straightforward way to implement the use of these extracts. Similarly, the use of aqueous plant extracts with additives that are already considered to be promising alternatives to traditional preservatives, such as nitrites and sulfites, should be also evaluated. Sodium acetate, for example, could be a promising candidate, since it has been demonstrated to inhibit *S. enterica* in a food model [112], as well as to reduce *Salmonella* biofilm formation [113].

Finally, an exhaustive study concerning the *in vivo* toxicity of the most promising extracts is a major challenge for their use in *Salmonella* infections management.

## Supplementary Materials

Supplementary materials can be found at https://www.mdpi.com/1422-0067/20/4/940/s1. Table S1. Antimicrobial activity of aqueous plant extracts against *Salmonella* serovars (Studies published between 2006–2018), Figure S1. Diagram representing antibacterial activity of aqueous plant extracts on *Salmonella* serovars *in vitro* since 2006, expressed as minimum inhibitory concentration (MIC) and considering MIC values above 1 mg/mL as exclusion criteria.
