# Supplementary material for: Current Challenges and Perspectives for the Use of Aqueous Plant Extracts in the Management of Bacterial Infections: The Case-Study of Salmonella enterica Serovars
Source: Int J Mol Sci. 2019 Feb 21;20(4):940. doi: 10.3390/ijms20040940 (PMC6412809; doi:10.3390/ijms20040940)
Supplement: Supplementary file 1 [file ijms-20-00940-s001.pdf]

Table S1. Antimicrobial activity of aqueous plant extracts against *Salmonella* serovars (Studies published between 2006–2018).

Plant	Family	Part Used	Antimicrobial Assay	Concentration (mg/mL)	MIC (mg/mL)	Inhibited Microrganism	No inhibited Microrganism	Reference
Abarema cochliacarpos Gomes Barneby & J. W. Grimes	Mimosaceae	Stem	Microdilution method	0.005–0. 25	0.25	*S.* Choleraesuis	-	[48]
Abrus precatorius L.	Fabaceae	Seed	Microdilution method	0.156–6	N	-	*S.* Typhimurium	[11]
Abrus precatorius L.	Fabaceae	Leaf	Broth dilution method	10–50	40	*S.* Typhi	-	[7]
Abrus precatorius L.	Fabaceae	Root	Broth dilution method	10–50	50	*S.* Typhi	-	[7]
Abrus precatorius L.	Fabaceae	Seed	Broth dilution method	10–50	30	*S.* Typhi	-	[7]
Acacia burkei Benth.	Fabaceae	Bark	Broth dilution method	0.1–12	3	*S.* Typhimurium	-	[18]
Acacia leucophloea (Roxb.) Willd.	Fabaceae	Bark	Microdilution method	0.156–5	N	-	*S.* Typhimurium	[11]
Acacia salicina Lindl.	Fabaceae	Leaf	Microdilution method	0.0625–10	2.5	*S.* Typhimurium	-	[8]
Acacia salicina Lindl.	Fabaceae	Leaf	Microdilution method	0.0625–10	2.5	*S.* Enteriditis	-	[8]
Acanthospermum glabratum (DC.) Wild	Asteraceae	Whole plant	Broth dilution method	0.1–12	6	*S.* Typhimurium	-	[18]
Achillea membranacea (Labill.) DC.	Asteraceae	Leaf	Microdilution method	4–64	NA	-	*S.* Typhimurium	[9]
Achillea millefolium L.	Asteraceae	Flower	Microdilution method	3.125–100	3.125	*S.* Typhimurium	-	[29]
Achyranthes aspera L.	Amaranthaceae	Root	Microdilution method	0.156–5	N	-	*S.* Typhimurium	[11]
Acorus calamus L.	Acoraceae	Rhizome	Microdilution method	0.156–5	N	-	*S.* Typhimurium	[11]

Aegle marmelos (L.) Corrêa	Rutaceae	Leaf	Microdilution method	0.156–6	N	-	*S.* Typhimurium	[11]
Agave sisalana Perrine	Agavaceae	Leaf	Broth dilution method	2.5–40	20	*S.* Typhi	-<br>-	[12]
Ageratum conyzoides L.	Asteraceae	Whole plant	Microdilution method	0.156–5	4.0	*S.* Typhimurium	-	[11]
Alchemilla mollis	Rosaceae	Aerial part	Microdilution method	0.016–1	0.125	*S.* Typhimurium		[96]
Alchornea cordifolia Müll.Arg.	Euphorbiaceae	Leaf	Agar dilution method	2.5–150	NA	-	*S.* Typhi	[13]
Alchornea cordifolia Müll.Arg.	Euphorbiaceae	Leaf	Agar dilution method	2.5–150	NA	-	*S.* Enteriditis	[13]
Allium sativum	Amaryllidaceae	Bulb	Microdilution method	0.000062–0.064	0.00125–0.001	S. Typhimurium	-	[63]
Allium sativum	Amaryllidaceae	Bulb	Microdilution method	0.000062–0.064	0.0005	S. Lagos	-	[63]
Allium sativum	Amaryllidaceae	Bulb	Microdilution method	0.000062–0.064	0.00025–0.001	S. Anatum	-	[63]
Allium sativum	Amaryllidaceae	Bulb	Microdilution method	0.000062–0.064	0.001	S. Kentuchy	-	[63]
Alstonia boonei	Apocynaceae	Bark	Broth dilution method	0.2–712	0.8–128 (hot water); 64–712 (cold water)	S. Typhi		[19]
Alstonia scholaris (L.) R. Br.	Apocynaceae	Leaf	Microdilution method	0.156–6	N	-	*S.* Typhimurium	[11]
Ammannia baccifera L.	Lythraceae	Whole plant	Microdilution method	0.156–5	N	-	*S.* Typhimurium	[11]
Andrographis paniculata (Burm.f.) Wall. ex Nees	Polygalaceae	Leaf	Microdilution method	0. 156–5	1	*S.* Typhimurium	-	[11]
Andrographis paniculata (Burm.f.) Wall. ex Nees	Polygalaceae	Stem	Microdilution method	0.156–5	1	*S.* Typhimurium	-	[11]
Anisophyllea laurina R.	Anisophylleaceae	Leaf	Agar dilution method	0.125–1	N		S. Typhi	[14]

Anisophyllea laurina R.	Anisophylleaceae	Stem bark	Agar dilution method	0.125–1	1	S. Typhi		[14]
Angiopteris evecta (G. Forst.) Hoffm.	Marattiaceae	Leaf	Microdilution method	0.156–5	N	-	*S.* Typhimurium	[11]
Anisopus mannii N.E.Br.	Asclepiadaceae	Stem	Broth dilution method	12.5–200	50	*S.* Gallinarium	-	[49]
Annona reticulata L.	Annonaceae	Leaf	Broth dilution method	0.001–10	0.1 (hot water); 10 (cold water)	*S.* Typhimurium	-	[15]
Annona reticulata L.	Annonaceae	Leaf	Microdilution method	0.156–5	NA	-	*S.* Typhimurium	[11]
Annona squamosa L.	Annonaceae	Leaf	Microdilution method	0.156–5	NA	-	*S.* Typhimurium	[11]
Anogeissus latifolia (Roxb. ex DC.) Wall. ex Guill. & Perr.	Combretaceae	Leaf	Microdilution method	0.156–5	1	*S.* Typhimurium	-	[11]
Anthocephalus chinensis (Lam.) A. Rich. ex Walp.	Rubiaceae	Leaf	Microdilution method	0.156–5	N	-	*S.* Typhimurium	[11]
Arctium minus Schkuhr	Asteraceae	Aerial part	Microdilution method	0.1875–1	NA	-	*S.* Typhimurium	[56]
Argemone mexicana L.	Papaveraceae	Leaf	Microdilution method	0.156–5	NA	-	*S.* Typhimurium	[11]
Aristolochia indica L.	Aristolochiaceae	Leaf	Microdilution method	0.156–5	NA	-	*S.* Typhimurium	[11]
Artemisia martima L.	Astereaceae	Aerial part	Broth dilution method	2–10	NA	-	*S.* Typhi	[17]
Artocarpus heterophyllus Lam.	Moraceae	Leaf	Macrodilution method	0.2–1	0.468	*S.* Typhimurium	-	[16]
Artocarpus heterophyllus Lam.	Moraceae	Leaf	Macrodilution method	0.2–1	0.488	*S. enterica*	-	[16]
Arum discoridis Sm.	Araceae	Leaf	Microdilution method	4–64	NA	-	*S.* Typhimurium	[9]
Arum hygrophilum Sm.	Araceae	Leaf	Microdilution method	4–64	4.0	*S.* Typhimurium	-	[9]

Azadirachta indica A. Juss.	Meliaceae	Leaf	Broth dilution method	2–10	NA	-	*S.* Typhi	[17]
Azadirachta indica A. Juss.	Meliaceae	Leaf	Microdilution method	0.156–5	0.25	*S.* Typhimurium	-	[11]
Bacopa monnieri (L.) Pennell	Plantaginaceae	Leaf	Microdilution method	0.156–5	NA	-	*S.* Typhimurium	[11]
Barleria prionitis L.	Acanthaceae	Leaf	Microdilution method	0.156–5	1.25	*S.* Typhimurium	-	[11]
Bauhinia purpurea L.	Fabaceae	Leaf	Microdilution method	0.156–5	NA	-	*S.* Typhimurium	[11]
Bauhinia variegata L.	Fabaceae	Leaf	Microdilution method	0.156–5	NA	-	*S.* Typhimurium	[11]
Bergenia ciliata (Haw.) Sternb.	Saxifragacea	Rhizome	Broth dilution method	2–10	<2	*S.* Typhi	-	[17]
Blechnum orientale L.	Blechnaceae	Leaf	Microdilution method	0.156–5	NA	-	*S.* Typhimurium	[11]
Boerhavia diffusa L. *nom. cons.*	Nyctaginaceae	Leaf	Microdilution method	0.156–5	NA	-	*S.* Typhimurium	[11]
Boerhavia difusa L. *nom. cons.*	Nyctaginaceae	Leaf	Agar dilution method	2.5–150	150	S. Typhi	-	[13]
Boerhavia difusa L. *nom. cons.*	Nyctaginaceae	Leaf	Agar dilution method	2.5–150	150	-	*S.* Enteriditis	[13]
Boscia angustifolia A. Rich.	Capparaceae	Root	Broth dilution method	0.16–80	NA	-	*S.* Typhi	[53]
Brachylaena transvaalensis Hutch. ex E. Phillips & Schweick.	Asteraceae	Leaf	Broth dilution method	0.1–12	1.25	*S.* Typhimurium	-	[18]
Bridelia micrantha (Hochst.) Baill.	Phyllanthaceae	Leaf	Agar dilution method	2.5–150	NA	-	*S.* Typhi	[13]
Bridelia micrantha (Hochst.) Baill.	Phyllanthaceae	Leaf	Agar dilution method	2.5–150	NA	-	*S.* Enteriditis	[13]
Bryophyllum pinnatum	Crassulaceae	Leaf	Agar diffusion method	4–512	NA		*S.* paratyphi	[19]

Bryophyllum pinnatum (Lam.) Kurz	Crassulaceae	Stem	Agar dilution method	6.25–50.	9.98	*S.* Typhi	-	[50]
Buchanania lanzan Spreng.	Anacardiaceae	Leaf	Microdilution method	0.156–5	NA	-	*S.* Typhimurium	[11]
Butea monosperma (Lam.) Taub.	Fabaceae	Flower	Microdilution method	0.156–5	NA	-	*S.* Typhimurium	[11]
Butea superba Roxb.	Fabaceae	Leaf	Microdilution method	0.156–5	NA	-	*S.* Typhimurium	[11]
Calotropis procera (Aiton) W.T.Aiton 1811	Apocynaceae	Leaf	Broth dilution method	2–10	NA	-	*S.* Typhi	[17]
Calotropis procera (Aiton) W.T.Aiton 1811	Apocynaceae	Leaf	Microdilution method	0.156–5	NA	-	*S.* Typhimurium	[11]
Canscora decussata (Roxb.) Schult	Gentianaceae	Whole plant	Microdilution method	0.156–5	NA	-	*S.* Typhimurium	[11]
Capsicum annuum L.	Solanaceae	Fruit	Broth dilution method	0.006–0.5	0.5	*S.* Typhimurium	-	[59]
Capsicum frutescens L.	Solanaceae	Fruit	Broth dilution method	0.006–0.5	0.5	*S.* Typhimurium	-	[59]
Careya arborea Roxb.	Lecythidaceae	Bark	Microdilution method	0.156–5	NA	-	*S.* Typhimurium	[11]
Carica papaya	Caricaceae	Leaf	Broth dilution method	0.2–712	128–712 (hot water); 128–712 (cold water)	*S.* Typhi		[19]
Cassia fistula L.	Fabaceae	Leaf	Microdilution method	0.156–5	0.625	*S.* Typhimurium	-	[11]
Cassia tora L.	Fabaceae	Leaf	Microdilution method	0.156–5	NA	-	*S.* Typhimurium	[11]
Cassia tora L.	Fabaceae	Leaf	Broth dilution method	0.2–712	0.8–712 (hot water); 64–712 (cold water)	*S.* Typhi		[19]
Catharanthus roseus L.	Apocynaceae	Root	Broth dilution method	0.1–12	4	*S.* Typhimurium	-	[18]
Catharanthus roseus L.	Apocynaceae	Leaf	Microdilution method	0.156–5	NA	-	*S.* Typhimurium	[11]

Cedrela toona Roxb. ex Rottler & Willd.	Meliaceae	Leaf	Broth dilution method	2–10	NA	-	*S.* Typhi	[17]
Celastrus paniculatus Willd.	Celastraceae	Leaf	Microdilution method	0.156–5	5.0	*S.* Typhimurium	-	[11]
Centaurea nigra L.	Asteraceae	Aerial part	Microdilution method	0.1875–1	NA	-	*S.* Typhimurium	[56]
Centaurea scabiosa L.	Asteraceae	Aerial part	Microdilution method	0.1875–1	NA	-	*S.* Typhimurium	[56]
Centella asiatica (L.) Urban	Apiaceae	Whole plant	Microdilution method	0.156–5	2.5	*S.* Typhimurium	-	[11]
Chenopodium ambrosioides L.	Amaranthaceae	Whole plant	Broth dilution method	0.1–12	8	*S.* Typhimurium	-	[18]
Cirsium arverense (L.) Ten.	Asteraceae	Aerial part	Microdilution method	0.1875–1	NA	-	*S.* Typhimurium	[56]
Cirsium palustre (L.) Coss.	Asteraceae	Aerial part	Microdilution method	0.1875–1	NA	-	*S.* Typhimurium	[56]
Cirsium vulgare (L.) Coss.	Asteraceae	Aerial part	Microdilution method	0.1875–1	NA	-	*S.* Typhimurium	[56]
Cissampelos hirta Klotzsch	Menispermaceae	Whole plant	Broth dilution method	0.1–12	8	*S.* Typhimurium	-	[18]
Cissampelos pareira L.	Menispermaceae	Leaf	Microdilution method	0.156–5	1.0	*S.* Typhimurium	-	[11]
Cissus quadrangularis L.	Vitaceae	Stem	Microdilution method	0.156–5	NA	-	*S.* Typhimurium	[11]
Citrus aurantifolia	Rutaceae	Fruit	Broth dilution method	0–512	256	S. paratyphi		[19]
Citrus medica L.	Rutaceae	Leaf	Microdilution method	0. 156–6	N	-	*S.* Typhimurium	[11]
Clausena anisata (Willd.) Hook. f. ex Benth.	Rutaceae	Leaf	Agar dilution method	0.1–5	N	-	*S.* Typhimurium	[20]
Clausena anisata (Willd.) Hook. f. ex Benth.	Rutaceae	Bark	Agar dilution method	0.1–5	N	-	*S.* Typhimurium	[20]
Clerodendrum indicum (L.) Kuntze	Lamiaceae	Leaf	Microdilution method	0.156–5	NA	-	*S.* Typhimurium	[11]

Clerodendrum serratum (L.) Moon.	Lamiaceae	Leaf	Microdilution method	0.156–5	N	-	*S.* Typhimurium	[11]
Colebrookea oppositifolia Smith	Lamiaceae	Leaf	Broth dilution method	1–10	N	-	*S.* Typhi	[22]
Commelina benghalensis L.	Commelinaceae	Leaf	Microdilution method	0.156–5	NA	-	*S.* Typhimurium	[11]
Croton roxburghii Balak.	Euphorbiaceae	Leaf	Microdilution method	0.156–5	N	-	*S.* Typhimurium	[11]
Croton roxburghii Balak.	Euphorbiaceae	Bark	Microdilution method	0.156–5	1.25	*S.* Typhimurium	-	[11]
Curcuma amada Roxb.	Zingiberaceae	Rhizome	Microdilution method	0.156–5	NA	-	*S.* Typhimurium	[11]
Curcuma angustifolia Roxb.	Zingiberaceae	Rhizome	Microdilution method	0.156–5	N	-	*S.* Typhimurium	[11]
Curcuma aromatica Salisb.	Zingiberaceae	Rhizome	Microdilution method	0.156–5	NA	-	*S.* Typhimurium	[11]
Curcuma longa L.	Zingiberaceae	Whole plant	Broth dilution method	32–256	N	-	*S.* Paratyphi	[23]
Cymbopogon citratus (DC.) Stapf.	Poaceae	Leaf	Broth dilution method	32–256	N	-	*S.* Paratyphi	[23]
Cyperus rotundus L.	Cyperaceae	Aerial part	Agar dilution method	0.5–5	N	-	*S.* Typhimurium	[93]
Cyperus rotundus L.	Cyperaceae	Aerial part	Agar dilution method	0.5–5	N	-	*S.* Enteriditis	[93]
Dalbergia latifolia Roxb.	Fabaceae	Bark	Microdilution method	0.156–5	N	-	*S.* Typhimurium	[11]
Datura inoxia Mill.	Solanaceae	Leaf	Microdilution method	0.0005–1.024	NA	-	*S.* Typhi	[24]
Datura inoxia Mill.	Solanaceae	Stem	Microdilution method	0.0005–1.024	NA	-	*S.* Typhi	[24]
Datura inoxia Mill.	Solanaceae	Root	Microdilution method	0.0005–1.024	NA	-	*S.* Typhi	[24]
Datura metel L.	Solanaceae	Leaf	Microdilution method	0.156–5	NA	-	*S.* Typhimurium	[11]

Derris indica (Lam.) Been.	Fabaceae	Leaf	Microdilution method	0.156–5	2.0	*S.* Typhimurium	-	[11]
Diospyros melanoxylon Roxb.	Ebenaceae	Leaf	Microdilution method	0.156–5	2.5	*S.* Typhimurium	-	[11]
Diospyros melanoxylon Roxb.	Ebenaceae	Bark	Microdilution method	0.156–5	0.312	*S.* Typhimurium	-	[11]
Drynaria quercifolia (L.) J. Sm.	Polypodiaceae	Leaf	Microdilution method	0.156–5	N		*S.* Typhimurium	[11]
Ecballium elaterium (L.) A. Rich.	Cucurbitaceae	Leaf	Microdilution method	4–64	NA	-	*S.* Typhimurium	[9]
Elephantopus scaber L.	Asteraceae	Leaf	Microdilution method	0.156–5	N		*S.* Typhimurium	[11]
Emblica officinalis Gaertn.	Phyllanthaceae	Leaf	Microdilution method	0.156–5	0.625	*S.* Typhimurium	-	[11]
Eminium spiculatum (Blume) Schott	Araceae	Leaf	Microdilution method	4–64	NA	-	*S.* Typhimurium	[9]
Enhydra fluctuans Lour.	Asteraceae	Whole plant	Microdilution method	0.156–5	NA	-	*S.* Typhimurium	[11]
Erycibe paniculata Roxb.	Convolvulaceae	Leaf	Microdilution method	0.156–5	N		*S.* Typhimurium	[11]
Eryngium foetidum L.	Apiaceae	Leaf	Microdilution method	0.156–5	0.500	*S.* Typhimurium	-	[11]
Eryngium foetidum L.	Apiaceae	Stem	Microdilution method	0.156–5	N	-	*S.* Typhimurium	[11]
Eucalyptus globulus Labill.	Myrtaceae	Leaves	Broth dilution method	2–10	4	*S.* Typhi	-	[17]
Euphorbia hirta L.	Euphorbiaceae	Whole plant	Broth dilution method	25–100	50	*S.* Typhi	-	[62]
Euphorbia pulcherima (Willd. ex Klotzsch, 1834)	Euphorbiaceae	Flower	Macrodilution method	1–1000	100	*S.* Paratyphi	-	[25]
Euphorbia pulcherima (Willd. ex Klotzsch, 1834)	Euphorbiaceae	Stem	Macrodilution method	1–1000	1	*S.* Paratyphi	-	[25]

Euphorbia pulcherrima (Willd. ex Klotzsch, 1834)	Euphorbiaceae	Leaf	Macrodilution method	1.0–1000	100	*S.* Paratyphi	-	[25]
Euphorbia pulcherrima (Willd. ex Klotzsch, 1834)	Euphorbiaceae	Whole plant	Macrodilution method	1.0–1000	1	*S.* Typhi	-	[25]
Euphorbia pulcherrima (Willd. ex Klotzsch, 1834)	Euphorbiaceae	Whole plant	Macrodilution method	1.0–1000	1	*S.* Paratyphi	-	[25]
Euphorbia pulcherrima (Willd. ex Klotzsch, 1834)	Euphorbiaceae	Flower	Macrodilution method	1–1000	N	-	*S.* Typhi	[25]
Ficus abutilifolia (Miq.) Miq.	Moraceae	Leaf	Microdilution method	3.13–25	NA	-	*S.* Typhi	[26]
Ficus racemosa L.	Moraceae	Bark	Microdilution method	0.156–5	4.0	*S.* Typhimurium	-	[11]
Flacourtia indica (Burm. f.) Merr.	Salicaceae	Bark	Microdilution method	0.156–5	NA	-	*S.* Typhimurium	[11]
Flemingia nana Roxb.	Fabaceae	Leaf	Microdilution method	0.156–5	NA	-	*S.* Typhimurium	[11]
Foeniculum vulgare Mill.	Apiaceae	Leaf	Broth dilution method	0.01 – 1	NA	-	*S.* Gallinarum	[27]
Foeniculum vulgare Mill.	Apiaceae	Leaf	Broth dilution method	0.01–1	NA	-	*S.* Typhimurium	[27]
Garcinia livingstonei T. Anderson	Clusiaceae	Bark	Broth dilution method	0.1–12	2	*S.* Typhimurium	-	[18]
Gmelina arborea Roxb.	Lamiaceae	Leaf	Microdilution method	0.156–5	NA	-	*S.* Typhimurium	[11]
Gundelia tournefortii L.	Astereaceae	Leaf	Microdilution method	4–64	NA	-	*S.* Typhimurium	[9]
Gymnosporia senegalensis (Lam.) Loes.	Celastraceae	Leaf	Broth dilution method	0.1–12	8	*S.* Typhimurium	-	[18]

Helichrysum longifolium DC.	Asteraceae	Leaf	Agar dilution method	0.5–5	NA	-	*Salmonella* spp.	[28]
Helicteres isora L.	Malvaceae	Root	Microdilution method	0.156–5	1.25	*S.* Typhimurium	-	[11]
Heliotropium indicum L.	Boraginaceae	Leaf	Microdilution method	0.156–5	NA	-	*S.* Typhimurium	[11]
Hemidesmus indicus (L.) R.Br.	Apocynaceae	Leaf	Microdilution method	0.156–5	N	-	*S.* Typhimurium	[11]
Hemidesmus indicus (L.) R.Br.	Apocynaceae	Stem	Microdilution method	0.156–5	NA	-	*S.* Typhimurium	[11]
Hesperis matronalis L.	Brassicaceae	Stem	Microdilution method	3.125–100	3.125	*S.* Typhimurium	-	[29]
Hieracium pilosella L	Asteraceae	Leaf	Microdilution method	3.125–100	NA	-	*S.* Typhimurium	[29]
Hieracium pilosella L	Asteraceae	Stem	Microdilution method	3.125–100	3.125	*S.* Typhimurium	-	[29]
Hieracium pilosella L.	Asteraceae	Flower	Microdilution method	3.125–100	NA	-	*S.* Typhimurium	[29]
Holarrhena pubescens Wall. ex G. Don	Apocynaceae	Leaf	Microdilution method	0.156–5	0.312	*S.* Typhimurium	-	[11]
Hyptis suaveolens (L.) Poit.	Lamiaceae	Leaf	Microdilution method	0.156–5	NA	-	*S.* Typhimurium	[11]
Ichnocarpus frutescens (L.) W.T. Aiton	Apocynaceae	Leaf	Microdilution method	0.156–5	NA	-	*S.* Typhimurium	[11]
Indigofera cassioides Rottl. ex. DC.	Fabaceae	Leaf	Microdilution method	0.156–5	NA	-	*S.* Typhimurium	[11]
Indigofera serpentinicola Schrire	Fabaceae	Root	Agar diffusion method	0.01–110	11.3	*S. enterica*	-	[54]
Ipomoea pandurata (L.) G.F.W.Mey.	Convolvulaceae	Leaf	Microdilution method	3.125–100	3.125	*S.* Typhimurium	-	[29]
Ipomoea pandurata (L.) G.F.W.Mey.	Convolvulaceae	Flower	Microdilution method	3.125–100	NA	-	*S.* Typhimurium	[29]
Ixora pavetta Andr.	Rubiaceae	Leaf	Microdilution method	0.156–5	NA	-	*S.* Typhimurium	[11]

Jasminum abyssinicum Hochst. ex DC.	Oleaceae	Leaf	Broth dilution method	0.01–1	0.1	*S.* Gallinarum	-	[27]
Jasminum abyssinicum Hochst. ex DC.	Oleaceae	Leaf	Broth dilution method	0.01–1	0.01	*S.* Typhimurium	-	[27]
Jatropha gossypiifolia L.	Euphorbiaceae	Leaf	Microdilution method	0.156–5	NA	-	*S.* Typhimurium	[11]
Kalanchoe crenata	Crassulaceae	Leaf	Agar diffusion method	4–512	512	*S.* paratyphi		[19]
Kalanchoe pinnata (Lam.) Pers.	Crassulaceae	Leaf	Microdilution method	0.156–5	NA	-	*S.* Typhimurium	[11]
Krauseola mosambicina (Moss.) Pax & K. Hoffm.	Caryophyllaceae	Whole plant	Broth dilution method	0.1–12	6.67	*S.* Typhimurium	-	[18]
Lagerstroemia speciosa (L.) Pers.	Lythraceae	Leaf	Microdilution method	0.156–5	NA	-	*S.* Typhimurium	[11]
Lantana camara L.	Verbenaceae	Leaf	Microdilution method	0.156–5	5.0	*S.* Typhimurium	-	[11]
Lawsonia inermis L.	Lythraceae	Leaf	Microdilution method	0.156–5	NA	-	*S.* Typhimurium	[11]
Lawsonia inermis	Lythraceae	Leaf	Agar dilution method	20.65–660	80	*S.* Typhi		[30]
Lawsonia sp.	Lythraceae	Leaf	Broth dilution method	0.001–10	0.001 (cold water); 0.01 (hot water)	*S.* Typhimurium	-	[15]
Leonotis leonorus (L.) R. Br.	Lamiaceae	Leaf	Radial patterns on the agar plates	0.1–5	N	-	*S.* Pooni	[31]
Leonotis ocymifolia (Burm.f.) Iwarsson	Lamiaceae	Aerial part	Broth dilution method	0.01–1	1	*S.* Gallinarum	-	[27]
Leonotis ocymifolia (Burm.f.) Iwarsson	Lamiaceae	Aerial part	Broth dilution method	0.01–1	NA	-	*S.* Typhimurium	[27]
Libidibia ferrea (Mart. ex Tul.) L. P. Queiroz	Fabaceae	Bark	Broth microdilution method	0.03–10	5	*S.* Enteriditis	-	[46]

Linum usitatissimum L.	Linaceae	Leaf	Microdilution method	0.156–5	NA	-	*S.* Typhimurium	[11]
Lippia javanica (Burm.f.) Spreng.	Verbenaceae	Leaf	Broth dilution method	0.1–12	6	*S.* Typhimurium	-	[18]
Litsea glutinosa (Lour.) C.B.Rob.	Lauraceae	Leaf	Microdilution method	0.156–5	NA	-	*S.* Typhimurium	[11]
Lupinus varius L.	Fabaceae	Leaf	Microdilution method	4–64	NA	-	*S.* Typhimurium	[9]
Madhuca longifolia (Koenig) Macbride	Sapotaceae	Flower	Microdilution method	0.156–5	0.625	*S.* Typhimurium	-	[11]
Mallotus philippinensis (Lam.) Muell. Arg.	Euphorbiaceae	Leaf	Microdilution method	0.156–5	NA	-	*S.* Typhimurium	[11]
Mandragora autumnalis Bertol.	Solanaceae	Leaf	Microdilution method	4–64	NA	-	*S.* Typhimurium	[9]
Mangifera indica L.	Anacardiaceae	Leaf	Broth dilution method	0.1–12	N	-	*S.* Typhimurium	[18]
Mangifera indica L.	Anacardiaceae	Leaf	Microdilution method	0.156–5	NA	-	*S.* Typhimurium	[11]
Melia azedarach L.	Meliaceae	Leaf	Broth dilution method	0.1–12	4	*S.* Typhimurium	-	[18]
Melia azedarach L.	Meliaceae	Leaf	Broth dilution method	2 –10	NA	-	*S.* Typhi	[17]
Mentha longifolia (L.) Huds.	Lamiaceae	Leaf	Microdilution method	0.3–10	5	*S.* Typhi	-	[32]
Mentha piperita L.	Lamiaceae	Leaf	Broth dilution method	0.001–10	0.1(hot water); 1 (cold water)	*S.* Typhimurium	-	[15]
Mimusops elengi L.	Sapotaceae	Leaf	Microdilution method	0.156–5	N	-	*S.* Typhimurium	[11]
Momordica dioica Roxb. ex Willd.	Cucurbitaceae	Leaf	Microdilution method	0.156–5	N	-	*S.* Typhimurium	[11]
Morinda citrifolia L.	Rubiaceae	Leaf	Microdilution method	0.625–10	N	-	*S.* Typhi	[33]
Morinda citrifolia L.	Rubiaceae	Leaf	Microdilution method	0.156–5	NA	-	*S.* Typhimurium	[11]

Moringa peregrine (Forssk.) Fiori	Moringaceae	Leaf	Microdilution method	0.5–20	0.675	*S.* Typhi	-	[34]
Musa sapientum L.	Musaceae	Fruit	Broth dilution method	32–256	100	*S.* Paratyphi		[23]
Myrsine africana L.	Primulaceae	Leaf	Broth dilution method	0.01–1	NA	-	*S.* Gallinarum	[27]
Myrsine africana L.	Primulaceae	Leaf	Broth dilution method	0.01–1	NA		*S.* Typhimurium	[27]
Neolitsea chinensis (Gamble) Chun	Lauraceae	Bark	Broth dilution method	2 –10	NA	-	*S.* Typhi	[17]
Nigella sativa L.	Ranunculaceae	Seed	Broth dilution method	2–10	NA	-	*S.* Typhi	[17]
Nyctanthes arbor-tristis L.	Oleaceae	Leaf	Microdilution method	0.156–5	0.5	*S.* Typhimurium	-	[11]
Nyctanthes arbor-tristis L.	Oleaceae	Bark	Microdilution method	0.156–5	0.125	*S.* Typhimurium	-	[11]
Ocimum basilicum Lam.	Lamiaceae	Leaf	Broth dilution method	1–20	NA	-	S. Typhi	[21]
Ocimum basilicum Lam.	Lamiaceae	Bud	Broth dilution method	1–20	7	*S.* Typhi	-	[21]
Ocimum basilicum Lam.	Lamiaceae	Seed	Broth dilution method	1–20	NA	-	S. Typhi	[21]
Ocimum gratissimum	Lamiaceae	Leaf	Broth dilution method	0–200	100	*S.* Typhimurium		[36]
Ocimum sanctum L.	Lamiaceae	Leaf	Broth dilution method	0.001–10	10 (Both cold or hot water)	*S.* Typhimurium	-	[15]
Oroxylum indicum (L.) Benth. ex Kurz	Bignoniaceae	Bark	Microdilution method	0.156–5	N	-	*S.* Typhimurium	[11]
Ozoroa insignis Delile.	Anacardiaceae	Stem bark	Microdilution method	0–2.5	0.625	*S.* typhi		[36]
Paederia foetida L.	Rubiaceae	Leaf	Microdilution method	0.156–5	0.5	*S.* Typhimurium	-	[11]

Parapiptadenia rigida (Benth.) Brenan	Fabaceae	Bark	Broth microdilution method	0.03–10	5	*S.* Enteriditis	-	[46]
Paronychia argentea Lam.	Caryophyllaceae	Leaf	Microdilution method	4–64	NA	-	*S.* Typhimurium	[9]
Phyllanthus fraternus Webster	Euphorbiaceae	Whole plant	Microdilution method	0.156–5	NA	-	*S.* Typhimurium	[11]
Piper trioicum Roxb.	Piperaceae	Leaf	Microdilution method	0.156–5	NA	-	*S.* Typhimurium	[11]
Pistacia integerrima Stew. ex Brandis	Anacardiaceae	Bark	Agar dilution method	0–10	9	*S.* Typhi		[47]
Pleurostylia capensis Turcz (Loes)	Celastraceae	Bark	Microdilution method	0.39–25	12.5	*S. Typhimurium*		[37]
Pleurostylia capensis Turcz (Loes)	Celastraceae	Leaf	Microdilution method	0.39–25	N	*S. Typhimurium*		[37]
Pleurostylia capensis Turcz (Loes)	Celastraceae	Root	Microdilution method	0.39–25	6.25	*S. Typhimurium*		[37]
Plumbago zeylanica L.	Plumbaginaceae	Leaf	Microdilution method	0.156–5	NA	-	*S.* Typhimurium	[11]
Polyalthia cerasoides (Roxb.) Bedd.	Annonaceae	Leaf	Microdilution method	0.156–5	NA	-	*S.* Typhimurium	[11]
Polygonum hydropiper L.	Polygonaceae	Aerial part	Agar dilution method	0.002–0.512	0.128	*S.* Typhi	-	[109]
Psidium guajava L.	Myrtaceae	Leaf	Agar dilution method	0.16–20	5	*S.* Typhimurium	-	[45]
Psidium guajava L.	Myrtaceae	Leaf	Broth microdilution method	0.03–10	5	*S.* Enteriditis	-	[46]
Pterospermum acerifolium (L.) Willd.	Sterculiaceae	Leaf	Microdilution method	0.156–5	0.625	*S.* Typhimurium	-	[11]
Punica granatum L.	Punicaceae	Fruit	Broth dilution method	2–10	8.0	*S.* Typhi	-	[17]

Punica granatum L.	Punicaceae	Fruit peel	Broth dilution method	104–3500	<104	*S.* Typhimurium		[94]
Punica granatum L.	Punicaceae	Fruit aril	Broth dilution method	104–3500	105	*S.* Typhimurium		[94]
Punica granatum L.	Punicaceae	Root	Microdilution method	0–2.5	0.156	*S.* Typhi		[36]
Rauvolfia serpentine (L.) Benth. ex Kurz	Apocynaceae	Root	Microdilution method	0.156–5	NA	-	*S.* Typhimurium	[11]
Ricinus communis L.	Euphorbiaceae	Leaf	Microdilution method	0.156–5	NA	-	*S.* Typhimurium	[11]
Rosa multiflora Thunb.	Rosaceae	Flower	Microdilution method	3.125–100	3.125	*S.* Typhimurium	-	[29]
Rosa multiflora Thunb.	Rosaceae	Leaf	Microdilution method	3.125–100	NA	-	*S.* Typhimurium	[29]
Rumex ecklonianuns Meissner	Polygonaceae	Whole plant	Radial patterns on the agar plates	0.1–5	NA		*S. pooni*	[19]
Rumex crispus L.	Polygonaceae	Whole plant	Broth dilution method	0.0039–0.5	NA	-	*S.* Typhimurium	[95]
Ruta chalepensis L.	Rutaceae	Leaf	Microdilution method	4–64	NA	-	*S.* Typhimurium	[9]
Saraca asoca (Roxb.) Wilde	Fabaceae	Leaf	Microdilution method	0.048–10	NA	-	*S.* Typhimurium	[38]
Saraca asoca (Roxb.) Wilde	Fabaceae	Leaf	Microdilution method	0.048–10	NA	-	*S.* Paratyphi	[38]
Saraca asoca (Roxb.) Wilde	Fabaceae	Leaf	Microdilution method	0.048–10	NA	-	*S.* Typhi	[38]
*Saraca asoca (*Roxb.) Wilde	Fabaceae	Leaf	Microdilution method	0.156–5	N	-	*S.* Typhimurium	[11]
Saraca indica L.	Fabaceae	Stem	Microdilution method	0.5–5	3	*S.* Typhimurium	-	[51]
Sarcostemma viminale (L.) R. Br	Apocynaceae	Stem	Broth dilution method	0.1–12	8	*S.* Typhimurium	-	[18]

Schleichera oleosa (Lour.) Merr.	Sapindaceae	Leaf	Microdilution method	0.156–5	N	-	*S.* Typhimurium	[11]
Schotia brachypetala Sond.	Fabaceae	Bark	Broth dilution method	0.1–12	2	*S.* Typhimurium	-	[18]
Sclerocarya birrea (A. Rich.) Hochst.	Anacardiaceae	Stem	Microdilution method	0.156–10	0.625	*S.* Typhimurium	-	[52]
Securidaca longipedunculata Fresen.	Polygalaceae	Root	Agar dilution method	16–250	16	*S.* Typhi	-	[55]
Securidaca longipedunculata Fresen.	Polygalaceae	Leaf	Broth dilution method	0.591–6.25	6.25	*S.* Typhi	.	[39]
Securidaca longipenduculata Fresen.	Polygalaceae	Leaf	Broth dilution method	0–200	NA	-	*S.* Typhi	[35]
Securideca longipedunculata Fresen.	Polygalaceae	Root	Broth dilution method	0.591–6.25	N	-	*S.* Typhi	[39]
Semecarpus anacardium L.f.	Anacardiaceae	Fruit	Microdilution method	0.156–5	0.50	*S.* Typhimurium	-	[11]
Senna occidentalis (L.) H.S. Irwin & R.C. Barneby	Fabaceae	Root	Broth dilution method	0.1–12	4	*S.* Typhimurium	-	[18]
Senna occidentalis (L.) H.S. Irwin & R.C. Barneby	Fabaceae	Leaf	Microdilution method	0.156–5	0.625	*S.* Typhimurium	-	[11]
Senna siamae (Lam.) H.S. Irwin & Barneby	Fabaceae	Leaf	Broth dilution method	0.5–20	1.0	*S.* Typhi	-	[40]
Shorea robusta L.	Dipterocarpaceae	Leaf	Broth dilution method	0–1.024	0.256–0.450	*S.* Typhi	-	[41]
Shorea robusta L.	Dipterocarpaceae	Leaf	Broth dilution method	0–1.024	0.7	*S.* Typhimurium	-	[41]
Shorea robusta Roth.	Dipterocarpaceae	Leaf	Microdilution method	0.156–5	NA	-	*S.* Typhimurium	[11]
Sida cordata (Burm.f.) Borssum	Malvaceae	Whole plant	Microdilution method	0.156–5	NA	-	*S.* Typhimurium	[11]

Silene virginica L.	Caryophyllaceae	Leaf	Microdilution method	3.125–100	NA	-	*S.* Typhimurium	[29]
Solanum nigrum L.	Solanaceae	Leaf	Radial patterns on the agar plates	0.1–5	N	-	*S.* Pooni	[31]
Solidago canadensis L.	Asteraceae	Leaf	Microdilution method	3.125–100	NA	-	*S.* Typhimurium	[29]
Sonchus asper L. (Hill).	Asteraceae	Aerial part	Microdilution method	0–2	NA	-	*S. typhi*	[56]
Spondias pinnata (L. f.) Kurz	Anacardiaceae	Leaf	Microdilution method	0.156–5	0.50	*S.* Typhimurium	-	[11]
Stachytarpheta jamaicensis	Verbenaceae	Leaf	Broth dilution method	0.2–712	0.8–128 (hot water); 64–712 (cold water)	*S.* Typhi		[19]
Strychnos madagascariensis Poir.	Loganiaceae	Leaf	Broth dilution method	0.1–12	8	*S.* Typhimurium	-	[18]
Syzygium aromaticum L.	Myrtaceae	Bud	Broth dilution method	0–2	0.0625–0.250	*S.* Typhi		[110]
Syzygium cordatum Hochst ex C. Krauss	Myrtaceae	Bark	Microdilution method	0 –2.5	0.156	*S.* Typhi		[36]
Syzygium cordatum Hochst ex C. Krauss	Myrtaceae	Bark	Broth dilution method	0.1–12	8	*S.* Typhimurium	-	[18]
Syzygium cumini (L.) Skeels	Myrtaceae	Leaf	Microdilution method	0.156–5	NA	-	*S.* Typhimurium	[11]
Syzygium jambos (L.) Alston	Myrtaceae	Leaf	Microdilution method	0.156–5	NA	-	*S.* Typhimurium	[11]
Tamarindus indica L.	Fabaceae	Fruit	Macrodilution method	7.81–125	62.5	*S.* Typhimurium	-	[60]
Tamarindus indica L.	Fabaceae	Fruit	Macrodilution method	7.81–125	31.25	*S.* Kitambo	-	[60]
Taraxacum officinale F.H. (Webb).	Asteraceae	Root	Microdilution method	0.1875–1	NA	-	*S.* Typhimurium	[56]
Tephrosia purpurea (L.) Pers.	Fabaceae	Fruit	Microdilution method	0.156–5	NA	-	*S.* Typhimurium	[11]

Terminalia alata Heyne ex Roth.	Combretaceae	Bark	Microdilution method	0.156–5	NA	-	*S.* Typhimurium	[11]
Terminalia arjuna (Roxb.) W. & A.	Combretaceae	Bark	Microdilution method	0.156–5	N	-	*S.* Typhimurium	[11]
Terminalia bellirica (Gaertn.) Roxb.	Combretaceae	Bark	Microdilution method	0.156–5	NA	-	*S.* Typhimurium	[11]
Terminalia chebula Retz.	Combretaceae	Bark	Microdilution method	0.156–5	NA	-	*S.* Typhimurium	[11]
Terminalia sericea Burch. ex DC.	Combretaceae	Bark	Broth dilution method	0.1–12	1	*S.* Typhimurium	-	[18]
Tribulus terrestris L.	Zygophyllaceae	Fruit	Broth dilution method	0.01–5	5	*S.* Typhimurium	-	[42]
Tribulus terrestris L.	Zygophyllaceae	Leaf	Broth dilution method	0.01–5	N	-	*S.* Typhimurium	[42]
Tribulus terrestris L.	Zygophyllaceae	Root	Broth dilution method	0.01–5	N	-	*S.* Typhimurium	[42]
Trichilia emetica Martin Vahl	Meliaceae	Bark	Broth dilution method	0.1–12	12	*S.* Typhimurium	-	[18]
Tridax procumbens L.	Asteraceae	Leaf	Microdilution method	0.156–5	N	-	*S.* Typhimurium	[11]
Trigonella Foenum-graecum	Fabaceae	Seed	Agar diffusion method	25–200	NA	-	*S.* Typhi	[57]
Urtica pilulifera L.	Urticaceae	Leaf	Microdilution method	4–64	NA	-	*S.* Typhimurium	[9]
Vaccinium oxycoccos	Ericaceae	Fruit	Macrodilution method	0.098–5	12.5	*S.* Enteriditis		[61]
Vaccinium oxycoccos	Ericaceae	Fruit	Macrodilution method	0.098–5	6.25	*S.* Typhimurium		[61]
Vangueria infausta Burch.	Rubiaceae	Bark	Broth dilution method	0.1–12	8	*S.* Typhimurium	-	[18]
Ventilago madraspatana Gaertn.	Rhamnaceae	Leaf	Microdilution method	0.156–5	NA	-	*S.* Typhimurium	[11]
Vernonia anthelmintica (L.) Willd.	Asteraceae	Leaf	Microdilution method	0.156 – 5	NA	-	*S.* Typhimurium	[11]

Vernonia natalensis Sch. Bip. ex Walp.	Asteraceae	Root	Broth dilution method	0.1–12	8	*S.* Typhimurium	-	[18]
Vitex doniana	Verbenaceae	Root	Broth dilution method	0.2–712	16–128 (hot water); 64–128 (cold water)	*S.* Typhi		[19]
Vitex negundo L.	Verbenaceae	Leaf	Microdilution method	0. 156–5	N	-	*S.* Typhimurium	[11]
Vitex negundo L.	Verbenaceae	Leaf	Agar dilution method	0.312–2.5	1.25	*S.* Typhimurium	-	[10]
Vitex negundo L.	Verbenaceae	Bark	Agar dilution method	0.312–2.5	0.625	*S.* Typhimurium	-	[10]
Vitis vinifera L.	Vitaceae	Leaf	Microdilution method	0.39–400	N	-	*S.* Typhimurium	[43]
Woodfordia fruticosa (L.) Kurz	Lythraceae	Leaf	Microdilution method	0.156–5	4.0	*S.* Typhimurium	-	[11]
Zataria multiflora Boiss.	Lamiaceae	Leaf	Microdilution method	0–0.1	N	-	*S.* Typhi	[44]
Zingiber officinale Roscoe	Zingiberaceae	Rhizome	Agar diffusion method	0.01–100	0.2	*S.* Typhi	-	[58]

N: Lack of inhibition of microbial growth at the maximum concentration of the extract/fraction used in the experiment, NA: extract is not active against bacteria. Microorganisms: *Salmonella enterica* serovar Typhi (*S.* Typhi), *Salmonella enterica* serovar Typhimurium (*S.* Typhimurium), *Salmonella enterica* serovar Enteriditis (*S.* Enteriditis), *Salmonella enterica* serovar Pooni (*S.* Pooni), *Salmonella enterica* serovar Paratyphi (*S.* Paratyphi), *Salmonella enterica* (*S. enterica*), *Salmonella enterica* serovar Gallinarum (*S.* Gallinarum), *Salmonella enterica* serovar Choleraesuis (*S.* Choleraesuis), *Salmonella enterica* serovar Kitambo (*S.* Kitambo).

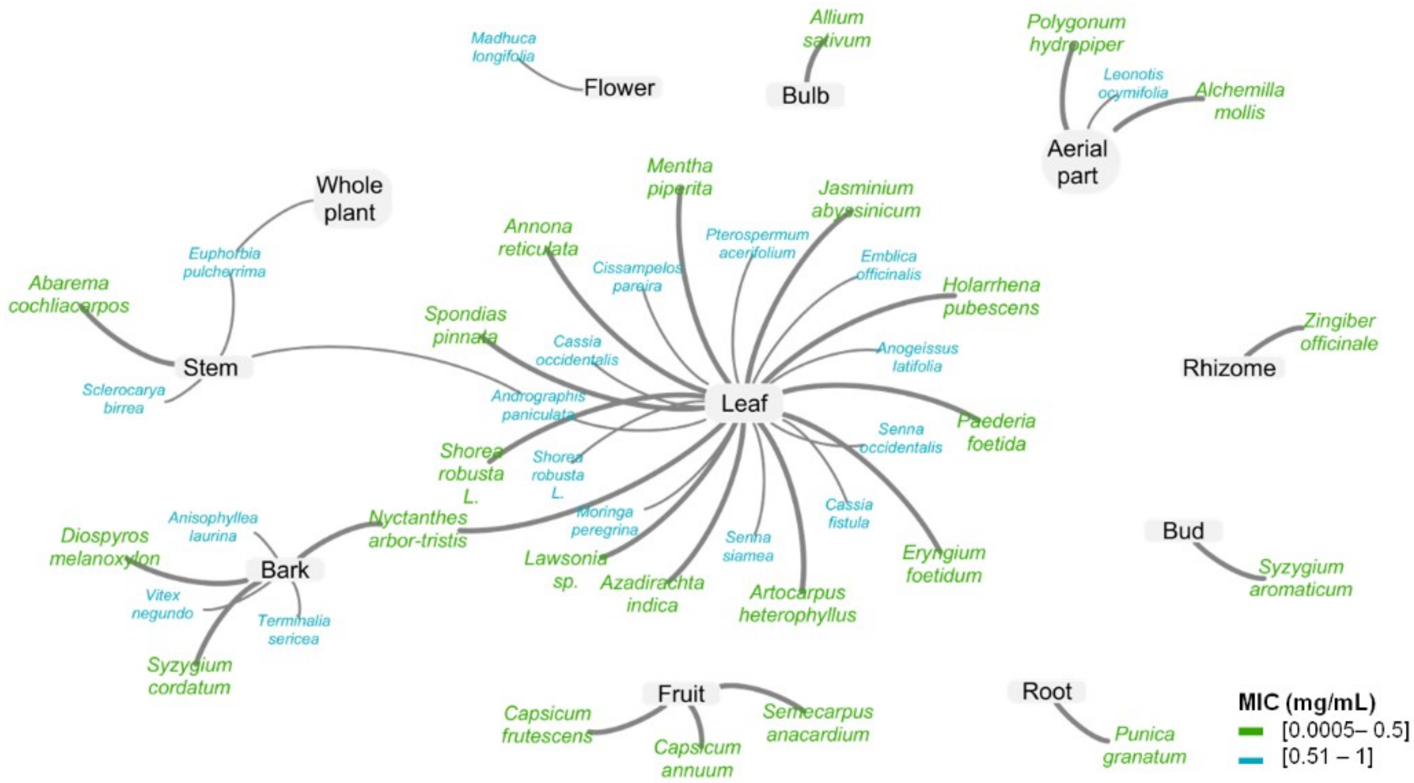

Figure S1. Diagram representing antibacterial activity of aqueous plant extracts on *Salmonella* serovars *in vitro* since 2006, expressed as minimum inhibitory concentration (MIC) and considering MIC values above 1 mg/mL as exclusion criteria (see Table S1). Grey lines connect the studies between each other through the colored nodes, which represent the plants part used, colored names represent names of plant species, and different colors/line widths represent different MIC ranges (see legend). As MICs increase the size of the letters of the plant species name get smaller.
